# Getting to the heart of Lewy body disease

**DOI:** 10.1172/JCI175798

**Published:** 2024-01-02

**Authors:** Anna E. Goodheart, Craig Blackstone

**Affiliations:** Department of Neurology, Massachusetts General Hospital and Harvard Medical School, Boston, Massachusetts, USA.

## Abstract

Early identification of neurodegenerative diseases before extensive neuronal loss or disabling symptoms have occurred is imperative for effective use of disease-modifying therapies. Emerging data indicate that central Lewy body diseases — Parkinson disease and dementia with Lewy bodies — can begin in the peripheral nervous system, opening up a therapeutic window before central involvement. In this issue of the *JCI*, Goldstein et al. report that cardiac ^18^F-dopamine positron emission tomography reveals lower activity selectively in individuals with several self-reported Parkinson disease risk factors who later develop Parkinson disease or dementia with Lewy bodies. Accurately identifying which at-risk individuals will develop central Lewy body disease will optimize early patient selection for disease-modifying therapies.

## Early nonmotor symptoms in Lewy body disease

Parkinson disease (PD) is a progressive neurodegenerative movement disorder with cardinal clinical features of rest tremor, muscular rigidity, bradykinesia (slowness of movement), and postural impairment. It afflicts an estimated 6.1 million people worldwide, an increase from approximately 2.5 million in 1990 (1), and the prevalence of PD is expected to continue to increase with the aging of the population in most developed countries. Pathologically, PD is characterized by intraneuronal Lewy body inclusions prominently containing misfolded α-synuclein, which normally functions as an intracellular trafficking protein, as well as prominent neuronal death of dopaminergic neurons in the midbrain substantia nigra pars compacta. The resulting disruption in dopamine neurotransmitter production and signaling in the basal ganglia circuit, the site of motor modulation, leads to the characteristic motor symptoms of PD. In the closely related but less common disease, dementia with Lewy bodies (DLB), Lewy bodies are found more prominently in the neocortex early in the disease, leading to presenting symptoms of dementia and visual hallucinations that are later accompanied by motor symptoms akin to those in PD (2). Due to their similar central nervous system pathologies, PD and DLB are grouped collectively as the central Lewy body diseases.

While clinical findings of PD and DLB are prominently associated with aberrant α-synuclein deposits in the central nervous system, pathologic α-synuclein can also be found in the peripheral nervous system. In fact, diagnosis of these disorders can be strongly supported by the identification of phosphosynuclein in dermal cutaneous nerve fibers present in skin punch biopsies (3). Also, single photon emission computed tomography (SPECT) scanning after intravenous injection of the sympathomimetic amine ^123^I-*meta*-iodobenzylguanidine (^123^I-MIBG) has demonstrated reduction in peripheral noradrenergic innervation of the heart in patients with PD (4). Importantly, it has been postulated that central α-synucleinopathies may begin in the peripheral nervous system before spreading to the central nervous system (5). Indeed, common early nonmotor symptoms in these disorders, such as orthostatic hypotension, constipation, and erectile dysfunction in men prefigure impairment of the peripheral and autonomic nervous systems rather than the brain. Anosmia or hyposmia can be a prodrome of PD, and α-synuclein can be found in cells of the olfactory bulb, suggesting that α-synuclein may originate in the environment and enter the olfactory system, and then extend into the brain and/or gut, whereby α-synuclein can then travel from the gastrointestinal tract upwards by way of the vagus nerve into the brainstem (5, 6). Despite these insights about the peripheral neurons, the main focus on central nervous system pathology and related symptoms drove the field for many years. An ability to identify patients at risk for developing central Lewy body disease would greatly facilitate the testing and implementation of disease-modifying therapies.

## Cardiac noradrenergic dysfunction as an early marker

In this issue of the *JCI,* Goldstein et al. (7) report the results of the prospective, longitudinal PDRisk study (ClinicalTrials.gov NCT00775853) investigating an NIH-developed positron emission tomography (PET) tracer, ^18^F-dopamine, to assess dysfunction of the cardiac noradrenergic system as a prelude to the development of PD and DLB. Though relatively small, this study demonstrated a remarkably accurate predictive value of low cardiac uptake of ^18^F-dopamine for central Lewy body disease. This finding was further corroborated by cerebrospinal fluid 3,4-dihydroxyphenylacetic acid (DOPAC), which the authors had previously demonstrated is low in individuals with preclinical PD as well as lower ^18^F-DOPA signal in the putamen, a marker of central nervous system dopaminergic terminal loss. As noted earlier, ^18^F-dopamine is not the first tracer to demonstrate utility in identifying cardiac sympathetic dysfunction, and these authors and others previously published pioneering cardiac imaging studies in patients with PD for decades using ^123^I-MIBG (4). However, Goldstein et al. are the first to our knowledge to demonstrate this relationship in a longitudinal, long-term prospective study of cardiac noradrenergic imaging in individuals with specific, self-reported nonmotor risk factors for PD (genetics, olfactory changes, dream enactment, orthostasis). Goldstein et al. (7) should pave the way for its use in identifying patients with peripheral synucleinopathies before they develop substantial central nervous system disease, thus optimizing the use of disease-modifying therapeutics.

## Timing of therapeutic interventions

At this time, there are no interventions that can convincingly prevent the development or progression of the central Lewy body diseases, although this is a very active area of investigation (8). Instead, treatment for both PD and DLB relies mostly on symptomatic therapies, typically replacing or augmenting the progressively declining levels of the neurotransmitter dopamine (9). However, the amount of dopamine replacement needed increases as the disease progresses, and at a certain point adverse effects of these therapies such as motor fluctuations, cognitive impairment, and dyskinesias can become debilitating and dose limiting. In this circumstance, an additional therapeutic option is neuromodulation. This process involves magnetic resonance–guided focused ultrasound or deep brain stimulation, in which electrodes are implanted into the basal ganglia or substantia nigra, with electrical stimulation of the motor circuitry adjusted to alleviate some motor symptoms. However, this latter approach requires invasive surgery and can be risky in an older population with medical comorbidities. Additionally, these therapies do not alleviate many symptoms of the disease, and the underlying neurodegeneration itself continues to progress at an unforgiving rate, ultimately overcoming the ability of neuromodulation to ameliorate disability and morbidity.

It is estimated that 50% to 80% of dopaminergic neurons in the substantia nigra are lost by the time noticeable clinical symptoms appear in PD. Unfortunately, this means that by the time clinical symptoms of neurodegeneration appear, it may be too late to meaningfully intervene. This challenge has stalled development of disease-modifying therapies for neurodegenerative diseases for decades because the ability to detect the very beginning of a neurodegenerative process has been elusive. Due to its early hypothesized involvement, the peripheral nervous system presents an attractive target for developing methods of detecting α-synuclein pathology early, before the onset of widespread central nervous system damage.

## Future implications

Goldstein et al. (7) demonstrate that a combination of cardiac noradrenergic cell loss and inefficient sequestration of catecholamines in residual cardiac sympathetic nerves precedes the onset of central Lewy body diseases, at least in a population with several identified nonmotor PD risk factors. The precise timing of cardiac noradrenergic cell loss to central nervous system dopaminergic cell loss remains to be elucidated. As the authors acknowledge, this paradigm may be more effective in identifying preclinical disease in patients with “body-first” rather than “brain-first” central Lewy body disease (10), since the choice of nonmotor risk factors for entry into the PDRisk study probably biases toward the “body-first” paradigm (7). Regardless, there is an increasing role for cardiac ^18^F-dopamine PET in identifying early or clinically prodromal disease in a substantial proportion of patients. Unfortunately, limited access to, and familiarity with, cardiac ^18^F-dopamine PET and the techniques used for result validation in this study currently hampers generalizability of this powerful approach. Hopefully, this study will spur others to obtain access to this ligand and develop the requisite expertise.

More broadly, the application of such a detailed and thorough longitudinal, prospective protocol as that used in Goldstein et al. (7), with highly compelling results, will stimulate not only an extension of this study but also the further development of available biomarker detection tools (11). For example, ^123^I-MIBG SPECT, skin and gut α-synuclein immunofluorescence, or seed amplification assays from cerebrospinal fluid or nasal secretions also have the potential to identify prodromal or early disease in at-risk individuals, accelerating the development and testing of new disease-modifying therapies for the central Lewy body diseases.

## Version 1 01/02/2024

Electronic publication
